# Effect of extra cysteine residue of new mutant 1Ax1 subunit on the functional properties of common wheat

**DOI:** 10.1038/s41598-017-07541-w

**Published:** 2017-08-08

**Authors:** Miao Li, Yaqiong Wang, Fengyun Ma, Jian Zeng, Junli Chang, Mingjie Chen, Kexiu Li, Guangxiao Yang, Yuesheng Wang, Guangyuan He

**Affiliations:** 1 The Genetic Engineering International Cooperation Base of Chinese Ministry of Science and Technology, The Key Laboratory of Molecular Biophysics of Chinese Ministry of Education, College of Life Science and Technology, Huazhong University of Science & Technology, Wuhan, 430074 China; 2 Department of Horticulture and Crop Science, The Ohio State University, 1680 Madison Avenue, Wooster, OH 44691 USA

## Abstract

Subunit pair 1Dx5 + 1Dy10 was recognized as superior subunit combination in wheat and contained an extra repetitive-domain cysteine residue in 1Dx5 that was important for understanding the formation of dough viscoelasticity. In this research, one specific serine codon of the *1Ax1* gene corresponding to the extra cysteine residue of 1Dx5 was substituted by a cysteine codon through site-directed mutagenesis. Four homozygous transgenic lines (T_4_) expressing the mutant *1Ax1* gene (*mut1Ax1*) were produced. Their greater dough strength and stability were confirmed by mixograph and were associated with highly increased gluten index, larger amounts of gluten macropolymers, larger size distribution for glutenin macropolymer particles and varied sodium-dodecyl-sulfate sedimentation volumes, compared with those of the one line expressing wild *1Ax1* that had similar expression level of transgene. The contents of β-sheets in dough and disulfide groups in gluten of the *mut1Ax1* transgenic lines were significantly increased. The microstructure of dough mixed to peak showed a more continuous gluten matrix in the mutant transgenic lines than the one line mentioned-above. It was concluded that the extra cysteine residue of mutant 1Ax1 subunit plays a positive role in contributing to dough strength and stability of wheat by cross-linking into gluten aggregates through inter-chain disulfide bonds.

## Introduction

The unique viscoelasticity of wheat dough is conferred by decisive gluten proteins in wheat grains and produces different types of wheat end-use products, such as breads, cakes, and other flour-based foods^1, 2^. Gluten proteins consist mainly of polymeric glutenins and monomeric gliadins, which are considered to be the major determinants of dough elasticity and viscosity, respectively^3, 4^. High molecular weight glutenin subunits (HMW-GSs) were highly related to the dough rheological properties^5, 6^, especially the high-quality subunit 1Ax1 and subunit pair 1Dx5 + 1Dy10. It was further predicated that the superiority of subunit pair 1Dx5 + 1Dy10 over other subunit pairs may be due to the role of the extra cysteine residue in the repetitive domain of subunit 1Dx5 during dough formation^7–10^.

The application of gene transformation methods in wheat has made it possible to compare the effects of x-type HMW-GSs on wheat flour properties. The expression of the exogenous genes *1Dx5* and *1Ax1* had different impacts on flour functional properties. Over-expression of transgene *1Dx5* dramatically increased the amount of glutenin polymers and thus resulted in “over-strong” dough properties, which were not suitable for making bread^11^. Meanwhile, over-expression of transgene *1Ax1* resulted in a more modest increase in dough strength^12, 13^. The “over-strong” characteristics of transgene *1Dx5* might result from the greater amounts of large-sized glutenin polymers due to the extra cysteine residue, which could promote the glutenin network to be more compact^14, 15^. However, Leόn *et al*.^16^ reported that the expression of subunit genes *1Ax1* and *1Dx5* in the bread-making wheat cv. Anza had similar effects on improving rheological properties, in which the ratios of 1Ax1/HMW and 1Dx5/HMW were 25.2% and 20.3%, respectively. The different reports about the impact of subunit 1Dx5 on dough rheological properties could be attributed to the differences in wheat genetic backgrounds and in the expression levels of the *1Dx5* gene.

Moreover, the extra cysteine in specific x-type HMW-GSs such as 1Bx7.1 may be not as important as previously reported or even interfere with glutenin polymerisation, which further has negative effect on the dough mixing properties^17, 18^. Thus, the effect of the extra cysteine residue of x-type HMW-GSs on the functional properties of flour remains unknown.

Therefore, further research is needed to better understand the impact of the extra cysteine residue on wheat flour processing properties. Subunits 1Ax1 and 1Dx5 have different effects on wheat flour functional properties; in most cases, the former moderately increases the flour functional property, and the latter has a detrimental effect^11, 12^. While subunit 1Ax1 has four cysteine residues, 1Dx5 has five, with one extra cysteine residue in the repetitive domain. Subunit 1Ax1 always appears singly due to the gene silencing of the y-type subunit at the same *Glu-A1* locus in the wheat genome. In contrast, subunit 1Dx5 generally occurs with 1Dy10^19, 20^, and its effect might be influenced by the total amounts of both subunits, and thus the ratio of 1Dx5/1Dy10 and a balanced D-subunit composition^14, 21^.

In the present study, subunit 1Ax1 was selected as a mutational target to evaluate the effect of a specific extra cysteine residue close to the N-terminal repetitive domain of x-type HMW-GSs such as subunit 1Dx5 on wheat flour characteristics. The serine codon in the repetitive domain of 1Ax1 subunit that corresponds to the extra cysteine residue of subunit 1Dx5 was substituted by a cysteine codon through site-directed mutagenesis. Then, the resulting mutant gene (*mut1Ax1*) and wild-type *1Ax1* gene (*wt1Ax1*) were separately expressed in transgenic wheat lines. The impact of mutant 1Ax1 subunit (Mut1Ax1) on the flour rheological characteristics and its possible mechanism were analysed from the results of sodium-dodecyl-sulfate (SDS) sedimentation volume, gluten index analysis, mixograph, size exclusion-high performance liquid chromatography (SE-HPLC), glutenin macropolymer (GMP) particle size distribution, scanning electron microscopy (SEM), and fourier transform infrared spectroscopy (FT-IR), compared with those of the non-transformed and non-transgenic segregant lines and two positive control lines expressing *wt1Ax1*.

## Results

### Generation of transgenic plants and homozygous lines of wheat

**Figure 1:**
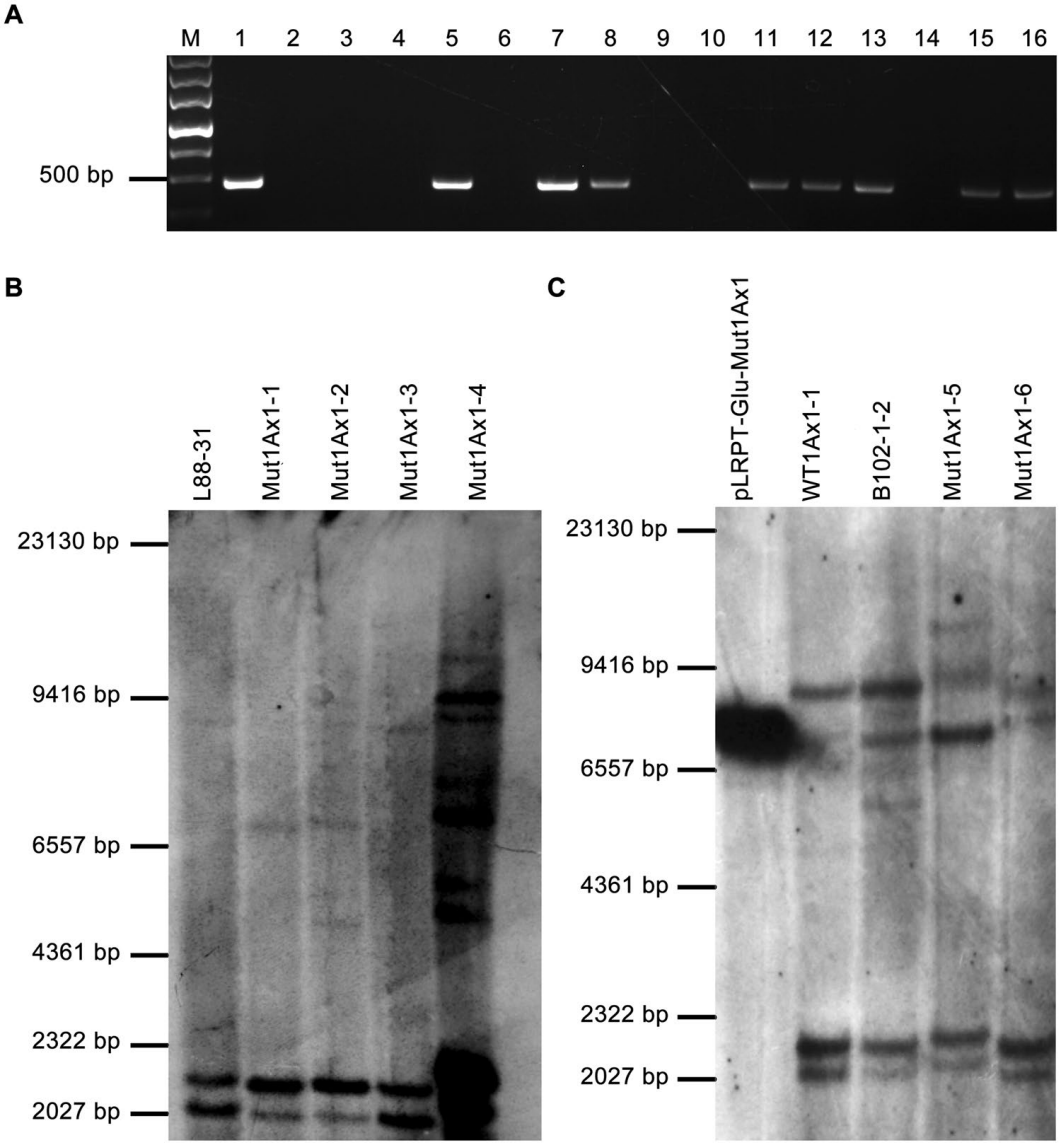
PCR (**A**) and Southern blotting analysis (**B** and **C**) of the transgenic wheat plants. Above: PCR amplification of the *CaMV35S* terminator sequence (**A**). Lane M: DNA Marker III; lane 1: pLRPT-Glu-Mut1Ax1 for positive control; lane 2: water for negative control; lane 3: genomic DNA of L88-31 for negative control; lanes 4-16: genomic DNA of regenerated wheat plants. Below: Southern blotting analysis (**B** and **C**) of *Bam*HI-cut genomic DNA from transgenic wheat lines (Mut1Ax1-1, -2, -3, -4, -5 and -6) and control lines (L88-31, WT1Ax1-1 and B102-1-2) probed with a 421 bp, DIG-labelled fragment of the pLRPT-Glu-Mut1Ax1 coding sequence. Positive control of pLRPT-Glu-Mut1Ax1 digested with *Bam*HI. The PCR results of transgenic lines expressing *mut1Ax1* in Fig. 1B and C are shown in lanes 5, 7, 8, 11, 12 and 13 of Fig. 1A, while that of line WT1Ax1-1 is shown in lane 15.

Approximately 1,000 immature wheat scutella were used for the bombardment of plasmid pLRPT-Glu-Mut1Ax1 and in turn for tissue differentiation and plant regeneration. Positive transgenic wheat plants were confirmed in the T_0_ generation by polymerase chain reaction (PCR) amplification (Fig. 1A). Meanwhile, transformed plants expressing the *wt1Ax1* gene were generated as described for those expressing the *mut1Ax1* gene. The sequencing results for the reverse transcription-PCR (RT-PCR) products of target gene *mut1Ax1* showed that the introduced gene expressed in the transgenic wheat lines had been successfully site-mutated (Supplementary Fig. S2C and D). Subsequently, the offspring of these transgenic T_0_ plants were identified based on Southern blotting analysis. As shown in Fig. 1B and C, these lines had different banding patterns that may be attributed to the rearrangement and/or truncation after transgene insertion^22, 23^; therefore, these lines were considered independent transgenic lines.

Four independent *mut1Ax1* transgenic lines and one independent *wt1Ax1* transgenic line were confirmed by sodium dodecyl sulfate-polyacrylamide gel electrophoresis (SDS-PAGE) to be homozygous with similar levels of transgene expression in the T_3_ generation: Mut1Ax1-1, Mut1Ax1–2, Mut1Ax1–5, Mut1Ax1–6 and WT1Ax1-1. The expression level of transgene *1Ax1* in line B102-1-2 was the highest. The abovementioned transgenic lines, the non-transgenic segregant line N-8 and line L88-31 were considered a set of isogenic wheat lines. A field experiment with these wheat lines with a randomised complete block design was carried out for subsequent analyses^24^.

### Agronomic performance of transgenic wheat lines

Transgenic wheat lines may have poorer agronomic characteristics with respect to their control lines^25, 26^. Here, as shown in Supplementary Table S1, there were no significant differences among all lines in traits such as heading date, number of spikelets per spike and 1,000-seed weight. There was no significant difference at the 0.01 probability level in growth period between transgenic and negative control lines (L88-31 and N-8) except for the positive control line B102-1-2; the number of seeds per spike was similar among lines, with the exception of transgenic line Mut1Ax1-6. The plant height of transgenic line Mut1Ax1-5 was significantly higher than that of control line N-8 (*P* < 0.01).

### Characterisation of storage proteins

**Figure 2:**
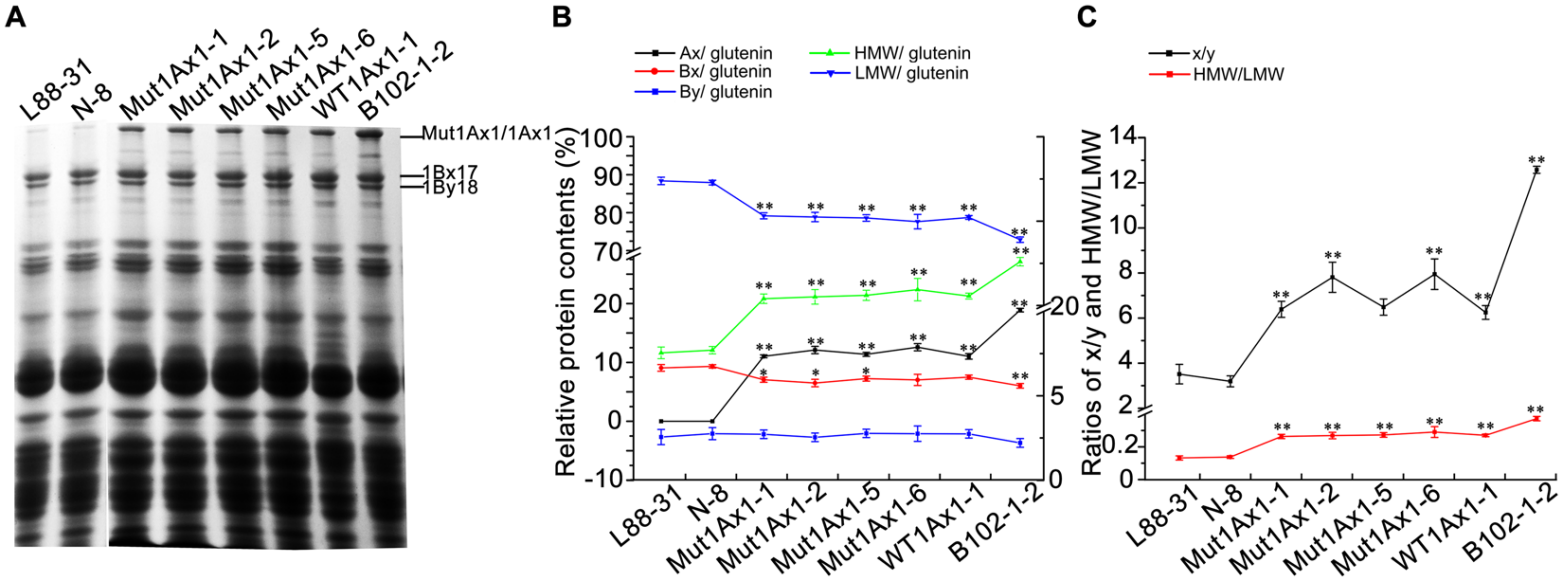
Characterisations of glutenin proteins in transgenic and control lines. (**A**) SDS-PAGE of seed proteins extracted from transgenic and control wheat lines. Transgenic Mut1Ax1 and endogenous subunit pair 1Bx17 + 1By18 were indicated by short lines on the right side of the gel. (**B**) The amounts and proportions of HMW-GS and LMW-GS and the ratios of x/y and HMW/LMW were determined by densitometry analysis for all lines. Data are given as the mean ± SEM (n = 4). * and ** show the comparisons between all transgenic lines and negative controls (L88-31 and N-8) (*P *<* 0.05, **P < 0.01 by Student’s t-test).

As shown in Fig. 2A, the transgenic subunit Mut1Ax1 or 1Ax1 was stably expressed in all transgenic wheat lines. Transgenic subunits accounted for 11%~13% of glutenin proteins in lines expressing *mut1Ax1* and line WT1Ax1-1 but approximately 19% in line B102-1-2. The total protein contents of the flours varied from 7.52% for the non-transformed control line L88-31 to 9.29% for the positive control line B102-1-2; this variation indicated a clear tendency for increases in the amount of total proteins in transgenic wheat lines.

The expression of *mut1Ax1* or *1Ax1* in the transgenic wheat lines altered the amounts and compositions of glutenin proteins in the endosperm, as shown in Supplementary Table S2 and Fig. 2. Relative to their negative controls L88-31 and N-8, all transgenic lines had higher contents of glutenin proteins and ratios of glutenin/gliadin. The glutenin fractions in flour increased from 3.53 μg mg^−1^ in line N-8 to 4.19 μg mg^−1^ in line B102-1-2. No significant difference in the contents of gliadin proteins was observed among lines. The glutenin/gliadin ratios in all transgenic wheat lines were higher than those in the two negative controls. Transgenic lines showed decreased contents of endogenous subunit 1Bx17 (Fig. 2B), but there was no significant difference among these lines in contents of subunit 1By18. However, all transgenic lines had higher significant increases in the ratios of x/y and HMW/LMW in glutenins (Fig. 2C).

Similar expression levels of *mut1Ax1* or *1Ax1* in transgenic lines except for B102-1-2 were observed. Compared with line WT1Ax1-1, the amount of glutenin proteins was highest in lines Mut1Ax1-1 (3.82 µg mg^−1^ flour), Mut1Ax1-2 (4.01 µg mg^−1^ flour), Mut1Ax1-5 (3.69 µg mg^−1^ flour) and Mut1Ax1-6 (4.07 µg mg^−1^ flour), and those of lines Mut1Ax1-2 and Mut1Ax1-6 were significantly higher (*P* < 0.01). Meanwhile, contents of gliadin proteins were slightly reduced in lines Mut1Ax1-1, -2 and -5 relative to line WT1Ax1-1. Moreover, the ratio of glutenin/gliadin ranged from 0.75 in line WT1Ax1-1 to 0.87 in line Mut1Ax1-1, with no significant difference (*P* < 0.01). Lines expressing *mut1Ax1* had slight increases in the amounts of Ax in glutenin proteins relative to line WT1Ax1-1 with no significant differences (Fig. 2B). In contrast, line WT1Ax1-1 had a higher proportion of subunit 1Bx17 in glutenins than did lines Mut1Ax1-1, -2, -5 and -6 without significant difference (*P* < 0.01). Based on the similar contents of 1By18 in glutenin proteins, lines expressing *mut1Ax1* and line WT1Ax1-1 had nearly equal amounts of HMW in glutenins. Additionally, all transgenic lines generated in this study had approximately 78% of low molecular weight glutenin subunit (LMW) proteins in glutenins. Overall, there were no significant differences on the ratios of x/y and HMW/LMW in glutenins (Fig. 2C).

Except for lines Mut1Ax1-1 and -5, the contents of gutenin proteins in the other lines, Mut1Ax1-2 and -6, were similar to those in line B102-1-2 (Supplementary Table S2). Additionally, there were no significant differences in the contents of gliadin proteins among lines. The ratio of glutenin/gliadin in the four *mut1Ax1* transgenic lines was 0.87, 0.86, 0.8 and 0.86, respectively; these values were lower than that in B102-1-2 (0.9) with no significant difference (*P* < 0.01). Line B102-1-2 had the characteristic of over-expression of transgene *1Ax1* (Fig. 2B). Compared with lines expressing *mut1Ax1*, the amounts of both endogenous subunits 1Bx17 and 1By18 in glutenins were lower in the positive control line B102-1-2 (Fig. 2B). In addition, the proportion of LMW proteins in glutenins of line B102-1-2 was significantly reduced with respect to those mutant transgenic lines. As a result, the ratios of x/y and HMW/LMW in line B102-1-2 were evidently higher than those in lines expressing *mut1Ax1* (Fig. 2C).

### Mixing property analysis

**Figure 3:**
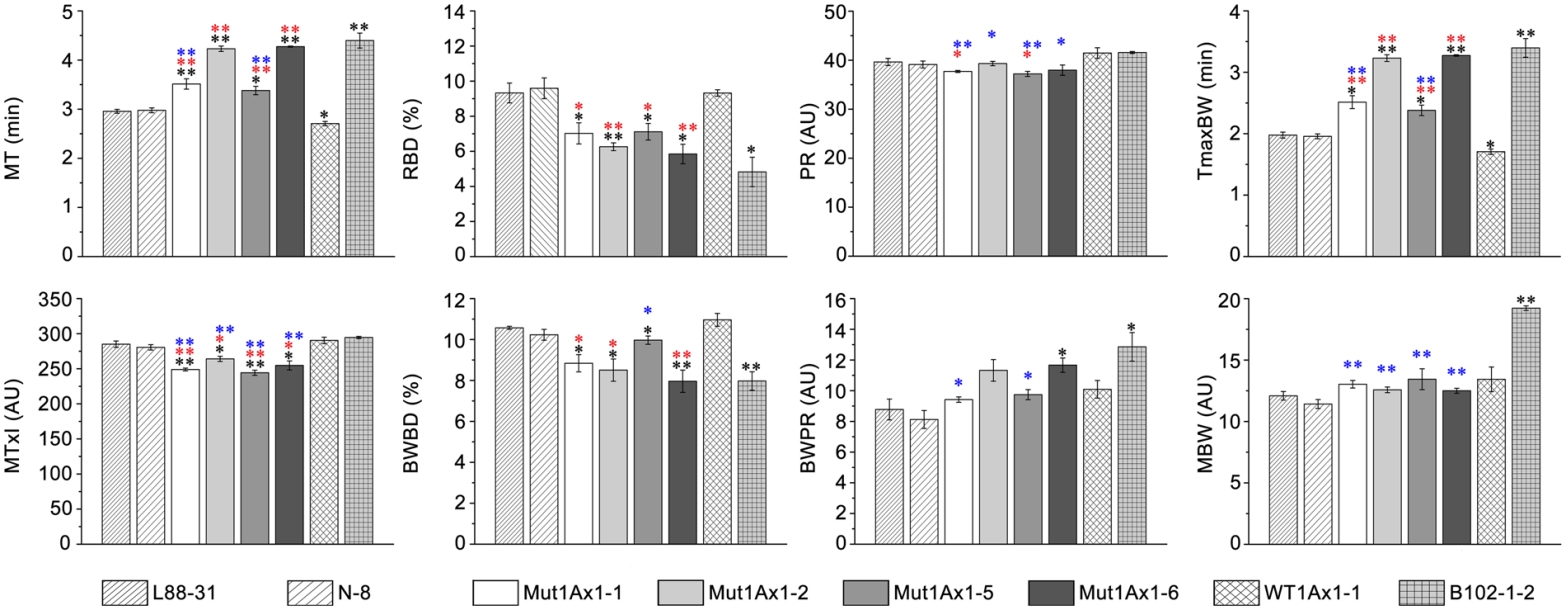
Effects of Mut1Ax1 on the dough mixing properties. Comparisons of the eight mixograph parameters among dough samples of all lines. Bars represent the mean ± SEM (n = 3). ^*/**^ Show the comparisons between all transgenic lines and negative controls (L88-31 and N-8) on the mixing parameters, while  and  represent comparisons between transgenic lines expressing *mut1Ax1* and line WT1Ax1-1 and comparisons between those lines and line B102-1-2, respectively (*P *<* 0.05, **P *<* 0.01 by Student’s t-test).

The dough mixing properties of control and transgenic wheat lines were determined using a 10-g mixograph with three replications. As seen in Fig. 3 and Supplementary Fig. S3, there were no significant differences among mixing parameters between the two negative control lines (N-8 and L88-31), which suggests that genetic transformation in the same background hardly had any effect on the dough mixing properties. All transgenic wheat lines differed from the two negative controls in the dough mixing properties, with the transgenic lines having longer mixing time (MT), higher bandwidth at peak resistance (BWPR) and maximum bandwidth during the mixing (MBW), and lower resistance to breakdown (RBD). These results indicated that the incorporation of subunit Mut1Ax1 or 1Ax1 improved the dough stability and its resistance to extension to some extent.

With respect to line WT1Ax1-1, the transgenic lines expressing *mut1Ax1* had significant increases in MT and time to maximum bandwidth (TmaxBW), which together with significant decreases in RBD and bandwidth breakdown (BWBD) showed greater dough strength and stability (Fig. 3). Although there was no significant difference in MBW between these lines, lines Mut1Ax1-2 and -6 had apparently higher values of BWPR, while the BWPR of the other two lines (Mut1Ax1-1 and -5) was similar to that of the positive control WT1Ax1-1. Dough resistance to extension was enhanced in lines Mut1Ax1-2 and -6.

As mentioned above, line B102-1-2 with a higher expression level of *1Ax1* and higher ratios of x/y and HMW/LMW, differed significantly from lines expressing *mut1Ax1*. Line Mut1Ax1-1 had no significant difference from the control in RBD and BWBD, exclusive of the other mixing parameters, while line Mut1Ax1-5 had no significant difference in RBD and BWPR compared with line B102-1-2. Whereas both lines Mut1Ax1-2 and -6 had no significant differences from line B102-1-2 among those mixing parameters, except for midline integral at 8 min (MTxI) and MBW (Fig. 3).

### Effects of Mut1Ax1 on gluten index and SDS-sedimentation volume

There were no significant differences in the gluten indexes or SDS-sedimentation volumes between the negative controls L88-31 and N-8 (Supplementary Table S2). Compared with the negative controls, gluten index increases were observed in all transgenic wheat lines with remarkably significant levels (*P* < 0.01) of Mut1Ax1-2 (59.47) and Mut1Ax1-6 (63.51) lines, significant levels (*P* < 0.05) of Mut1Ax1-1 (53.4) and Mut1Ax1-5 (53.7) and no significant level of WT1Ax1-1 (50.21). Compared with WT1Ax1-1, significant levels of Mut1Ax1-2 and Mut1Ax1-6 and no significant levels of Mut1Ax1-1 and Mut1Ax1-5 were observed in the transgenic lines. Line Mut1Ax1-6 was not significantly different from the control line B102-1-2 in this parameter. Meanwhile, the SDS-sedimentation volumes in all six transgenic lines were significantly higher than those of the two negative controls. The sedimentation values of all mutant transgenic lines were remarkably lower than those in line WT1Ax1-1. This result might be due to the formation of a more compact gluten matrix, which was promoted by the extra cysteine residue^21, 27^.

### Effects of Mut1Ax1 on the formation of gluten aggregates

**Figure 4:**
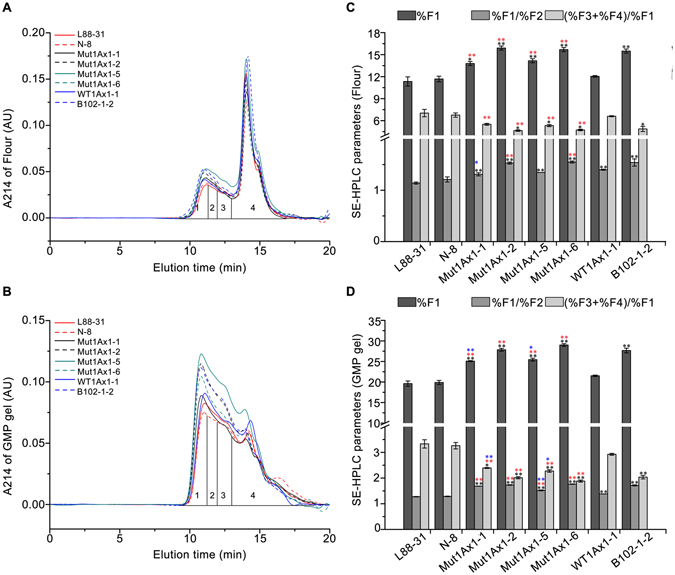
Effects of Mut1Ax1 on the formation of HMW aggregates. SE-HPLC analyses of flour and GMP gel samples of transgenic and control lines are shown in (**A**) and (**B**), with corresponding comparisons of three parameters (%F1, %F1/%F2, and (%F3 + %F4)/%F1) analysed statistically in (**C**) and (**D**). Bars represent the mean ± SEM (n = 4). */** show the comparisons between all transgenic lines and negative controls (L88-31 and N-8) on the three parameters above, while  and  represent comparisons between transgenic lines expressing *mut1Ax1* and line WT1Ax1-1 and comparisons between those lines and line B102-1-2, respectively (*P *<* 0.05, **P *<* 0.01 by Student’s t-test).

Gluten proteins were fractionated by SE-HPLC based on molecular masses without reducing the inter-chain disulfide bonds. Figure 4 shows typical HPLC profiles of fractions extracted from flour/GMP gel samples of control and transgenic wheat lines. These parameters from SE-HPLC profiles were associated with dough strength, with which the proportion of large-sized polymers (%F1), %F1/%F2 and (%F3 + %F4)/%F1 showed particularly strong correlations^28^.

The six transgenic wheat lines had obviously higher values of %F1, lower ratios of (%F3 + %F4)/%F1 and higher ratios of %F1/%F2 compared with the negative controls L88-31 and N-8 (Fig. 4). It could be concluded that transgenic subunits incorporated the formation of gluten polymer and increased the amounts of large-sized polymers.

Notably, the peaks at fractions 1 and 2 of lines expressing *mut1Ax1* were higher and accompanied by a left shift compared with those of line WT1Ax1-1 (Fig. 4A and B). In addition, the relative amount of large-sized polymers (%F1) was clearly higher in the lines expressing *mut1Ax1*. Moreover, the values of %F1/%F2 in lines Mut1Ax1-1, -2 and -6 significantly increased, while the (%F3 + %F4)/%F1 ratio in the four lines expressing *mut1Ax1* clearly decreased in flour/GMP gel samples (Fig. 4C and D). Furthermore, the size distribution of gluten proteins in lines expressing *mut1Ax1* with clearly lower transgene expression levels was similar to that of the positive control B102-1-2 with overexpression of the *1Ax1* gene (Fig. 4C). For GMP gels, there were no significant differences on the above three parameters between line B102-1-2 and the two of transgenic lines expressing *mut1Ax1* (Mut1Ax1-2 and -6).

### Effects of Mut1Ax1 on GMP particle size distribution

**Figure 5:**
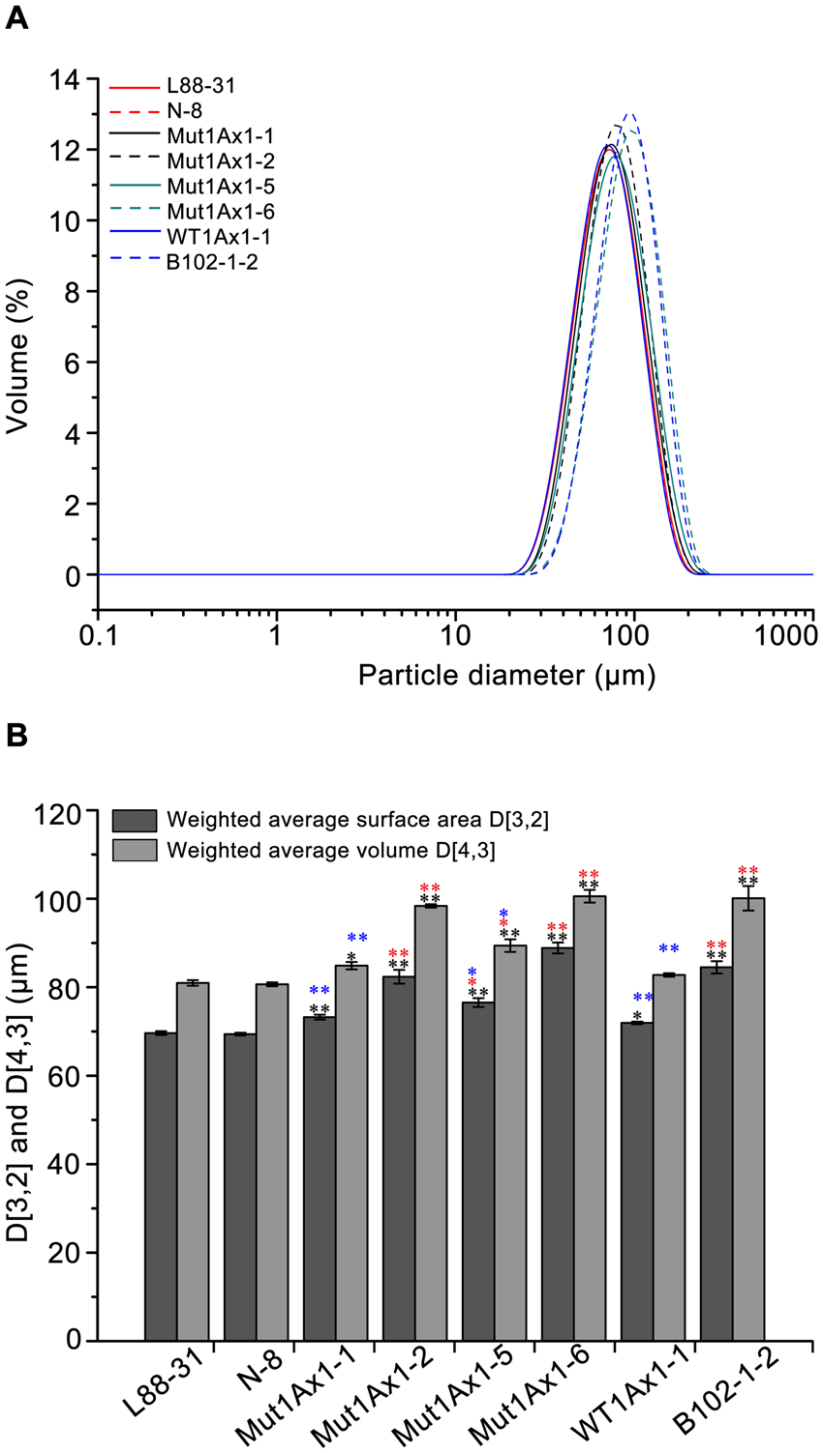
Effects of Mut1Ax1 on GMP particles size distribution. (**A**) The size distribution patterns of GMP particles extracted from defatted flour samples. (**B**) Weighted average diameters D[3,2] and D[4,3] of GMP particles. Bars represent the mean ± SEM (n = 4). */** Show the comparisons between all transgenic lines and negative controls (L88-31 and N-8) on the two parameters above, while  and  represent comparisons between transgenic lines expressing *mut1Ax1* and line WT1Ax1-1 and comparisons between those lines and line B102-1-2, respectively (*P *<* 0.05, **P *<* 0.01 by Student’s t-test).

GMP was comprised of large glutenin structures and thus correlated closely with the rheological property of dough^29, 30^. In all transgenic wheat lines except for WT1Ax1-1, the particle diameters of GMP became larger with an obvious right shift compared with negative controls L88-31 and N-8 (Fig. 5A). This result is consistent with the results of the weighted average diameters (Fig. 5B). With respect to D[3,2] and D[4,3], lines Mut1Ax1-2, -5, and -6 increased significantly relative to line WT1Ax1-1 (*P* < 0.01), while line Mut1Ax1-1 had a slight increase with no significant difference. The values of D[3,2] in lines expressing *mut1Ax1* were 73.23, 82.37, 76.53 and 88.87 μm respectively, while those of D[4,3] varied from 84.87 to 100.57 μm. There were no significant differences in the weighted average diameters between line B102-1-2 (84.5 μm and 100.1 μm) and lines Mut1Ax1-2 and -6.

### Effects of Mut1Ax1 on the amount of disulfide bonds

**Figure 6:**
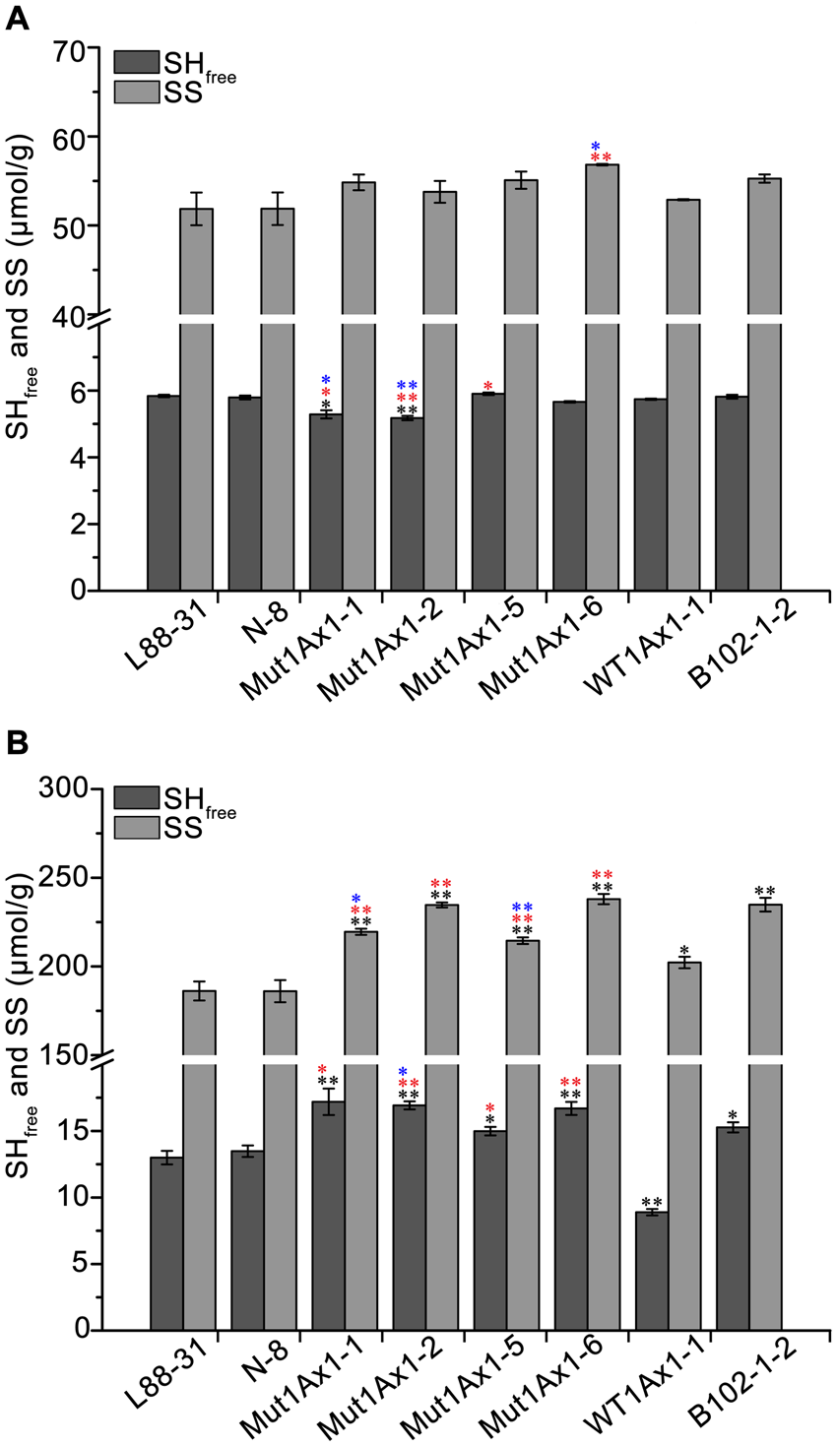
Comparisons of free thiol and disulfide groups in flours and glutens from transgenic and control wheat lines. (**A**) Relative contents of free thiol and disulfide groups in flour samples from all lines. (**B**) Relative contents of free thiol and disulfide groups in ground freeze-dried samples from all lines. Bars represent the mean ± SEM. At least three biological experiments were performed. */** Show the comparisons between all transgenic lines and negative controls (L88-31 and N-8) on the contents of free thiol and disulfide groups, while  and  represent comparisons between transgenic lines expressing *mut1Ax1* and line WT1Ax1-1 and comparisons between those lines and line B102-1-2, respectively (*P *<* 0.05, **P *<* 0.01 by Student’s t-test).

Thiol and disulfide contents are involved in the correct folding of seed storage proteins during grain development and are thus correlated with glutenin polymer conformation. The contents of free sulfhydryl (SH_free_) and disulfide (SS) in flour/ground freeze-dried gluten samples were investigated as described previously without thermomechanical treatment^31^. In comparisons with negative controls (L88-31 and N-8), the SS contents in flour samples were not significantly different in all transgenic lines, but the SS contents in gluten significantly increased (Fig. 6). The SS contents in flour samples of lines Mut1Ax1-1, -2, and -5 increased slightly relative to those of line WT1Ax1-1 with no significant difference. In contrast, the SS contents of line Mut1Ax1-6 were evidently higher than those of line WT1Ax1-1. In gluten samples, the contents of SH_free_ and SS in lines expressing *mut1Ax1* were significantly higher than those of line WT1Ax1-1 (*P* < 0.01) (Fig. 6B). Line B102-1-2 with higher expression of *1Ax1* was not significantly different from lines Mut1Ax1-1, -2 and -5 in the SS contents in flour, and it did not differ from lines Mut1Ax1-2 and -6 in the SS contents in gluten samples.

### Effects of Mut1Ax1 on the microstructure of dough

**Figure 7:**
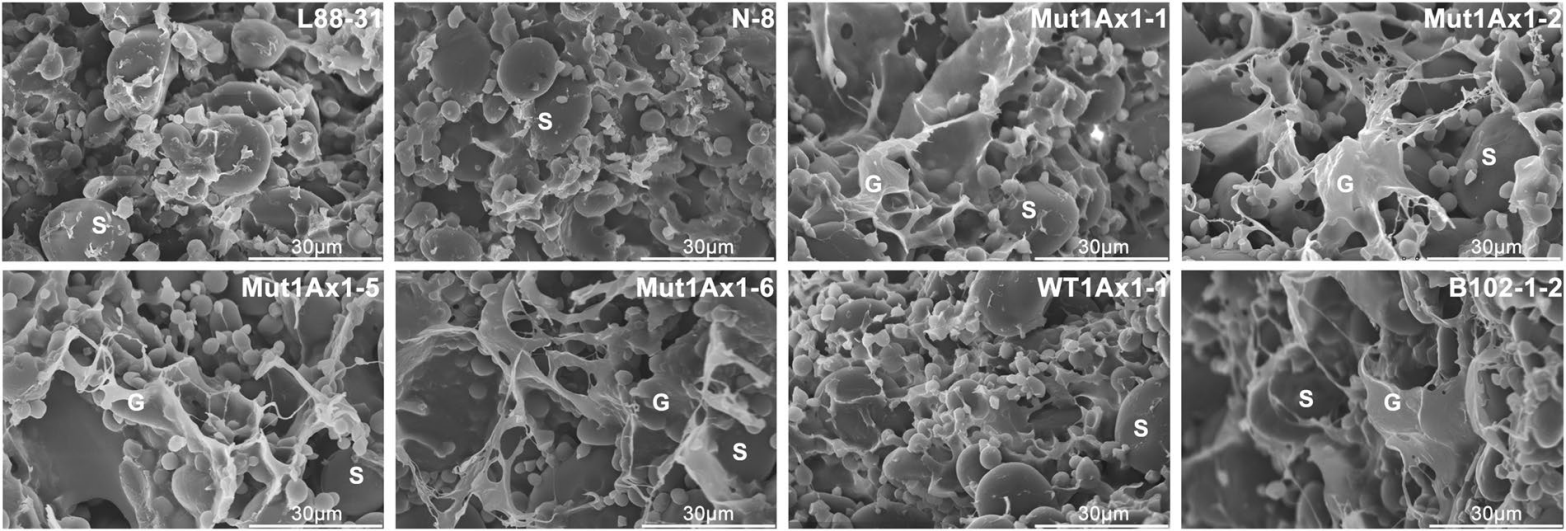
Effects of Mut1Ax1 on the microstructure of dough mixed to peak by SEM. Displays of the starch granules (S) and the gluten network structures (G).

SEM was performed to determine the effects of the Mut1Ax1 subunit on the microstructure of wheat dough mixed to peak. Obvious distinctions from imagines are shown in Fig. 7. In the two negative lines L88-31 and N-8, the starch granules were surrounded by discrete gluten matrix. Conversely, in all transgenic wheat lines except for WT1Ax1-1, a more continuous and apparent structure was observed. Compared with the line WT1Ax1-1, the structure of the gluten network was more visible in all lines expressing *mut1Ax1*. At the same time, similar amounts of network matrix were observed in lines expressing *mut1Ax1* and the control line B102-1-2.

### Effects of Mut1Ax1 on protein secondary structure

**Figure 8:**
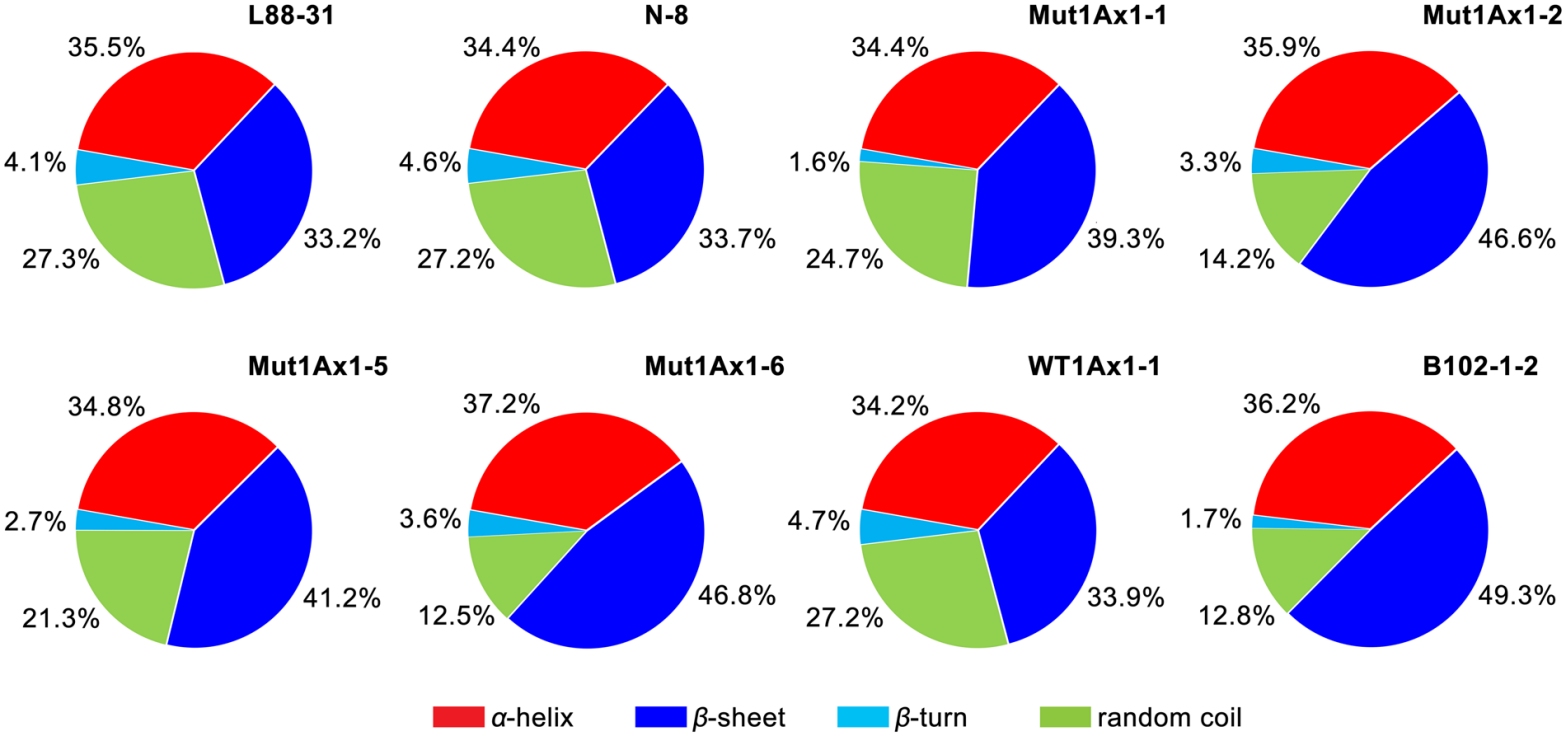
Effects of Mut1Ax1 on the secondary structure of gluten revealed by FT-IR. Values are given as the mean percentage of each secondary structure (n = 4).

The curve-fitting result of dough mixed to peak is shown in Supplementary Fig. S4 to demonstrate the conformation changes of gluten proteins based on the introgression of the *mut1Ax1* gene. Typical protein bands were observed over the region of 1,350-1,200 cm^−1^. After curve-fitting analysis, the α-helix (~1,314 cm^−1^) and β-sheet (~1,240 cm^−1^) structures occupied larger proportion of the total area (Fig. 8 and Supplementary Fig. S4), which is similar to previously reported results^32^. Lines expressing *mut1Ax1* had no remarkable differences in the α-helix content compared with the control line WT1Ax1-1 under similar conditions. There were significant increases in the β-sheet content of lines expressing *mut1Ax1* (from 39.3% to 46.8%) relative to line WT1Ax1-1 (33.9%). Meanwhile, a striking decrease in the band at ~1,284 cm^−1^ was observed in dough samples of lines expressing *mut1Ax1*; this decrease corresponded to the random coil conformation. Moreover, the β-turn content was 1.6% in line Mut1Ax1-1, which was significantly lower than 4.7% in line WT1Ax1-1, and slightly decreased in lines Mut1Ax1-2, -5 and -6.

Furthermore, the α-helix content in lines expressing *mut1Ax1* was not significantly different from that of the control line B102-1-2. Except for lines Mut1Ax1-1 and -5, there were no significant differences in the β-sheet and random coil contents between B102-1-2 and two transgenic lines expressing *mut1Ax1* (Mut1Ax1-2 and -6). In contrast, a distinct difference was observed: the β-turn contents were 3.3% (Mut1Ax1-2), 2.7% (Mut1Ax1-5), and 3.6% (Mut1Ax1-6), respectively. These values were evidently higher than that of the control B102-1-2 (1.7%).

## Discussion

The effects of the number and position of cysteine residues on the size of glutenin polymers and other flour functional properties have always been considered a hot research topic^17, 33^. The mechanism of the “over-strong” characteristics for subunit 1Dx5 with an extra cysteine residue remains unclear. Some researchers have attributed this mechanism to the presence of the extra cysteine residue in the repetitive domain^14^, which is due to the “head-to-tail” HMW polymer formed by disulfide crosslinking among HMW-GSs^34^. Some reports of subunit 1Dx5 without the extra cysteine residue have shown that the formation and stability of glutenin polymers might be due to the inter-chain hydrogen bonds presented in the conservative central repetitive domains^18^.

In our study, lines expressing *mut1Ax1* and line WT1Ax1-1 had similar transgene expression levels, which was important for comparing the relevant experimental results. The introgressions of transgenes *mut1Ax1* and *wt1Ax1* (WT1Ax1-1) led to a similar effect on glutenin protein characterisation (Fig. 2 and Supplementary Table S2). Interestingly, more gluten aggregates were formed in GMP gels of the mutant transgenic lines due to their highly significant increases in the amounts of large- and medium-sized polymers (Fig. 4B and D). This result agrees with the results of the gluten index and size distribution of GMP particles (Supplementary Table S2 and Fig. 5). All lines expressing *mut1Ax1* had obvious right shifts to a larger size distribution for GMP particles (Fig. 5), which helped to form a more compact gluten matrix. This change appears to be due to the extra cysteine residue forming highly cross-linked polymers relative to the control WT1Ax1-1. This residue could further lead to decreased SDS-sedimentation volumes of all mutant lines compared with the abovementioned control line^27, 35–37^. All mutant transgenic lines had significantly higher SDS-sedimentation volumes than those of the two negative controls (L88-31 and N-8), which was similar to the results in previous reports^21, 27, 38^. These data support our results of significant increases in the SS contents of gluten samples (Fig. 6). These results were further supported by the results of SEM and FT-IR analyses (Figs 7 and 8 and Supplementary Fig. S4). The more visible structure of the gluten network in dough of all mutant transgenic lines demonstrated that subunit Mut1Ax1 promoted the formation of protein network structure and thus positively impacted the dough quality. Secondary conformational changes of gluten proteins during mixing have been contributed to intermolecular interactions and are closely related to the rheological characteristics of wheat dough^32^. Significant increases in the β-sheet contents in these lines indicate that the amount of glutenin aggregations increased^39^, while relative decreases in the β-turn contents showed that intermolecular disulfide bonds were formed and promoted the conversion from β-turn to β-sheet. Compared with line WT1Ax1-1, the contents of random coils were significantly reduced in lines Mut1Ax1-2, -5 and -6 when the total β-sheet, β-turn and random coil contents were similar among the four transgenic lines. Based on the disulfide crosslinkage resulting from the extra cysteine residue, the incorporation of the Mut1Ax1 subunit might drive this transition from random coils to β-sheets and β-turns^39, 40^. All of the abovementioned results suggested that the extra cysteine residue in the repetitive domain was cross-linked into the formation of gluten aggregates and network matrix by intermolecular disulfide bonds. That implied that the superior dough properties of lines expressing *mut1Ax1* were due to the incorporation of the Mut1Ax1 subunit, which led to longer MT, lower RBD and higher BWPR in lines Mut1Ax1-2 and -6 with significant differences.

The SE-HPLC results of GMP gels indicated that the size distribution of gluten proteins between the positive control line B102-1-2 and lines Mut1Ax1-2 and -6 was similar (Fig. 4D). Additionally, the control differed from lines Mut1Ax1-2 and -6 in the SS contents in gluten samples with no significant difference (Fig. 6B). GMP gels was consisted mainly of HMW-GS and LMW-GS, which were cross-linked with interchain disulfide bonds^41^. It was then likely that subunit Mut1Ax1 could incorporate into glutenin polymers, enhancing the formation of gluten aggregates based on the extra cysteine residue. In addition, similar scales of network structure were observed in lines expressing *mut1Ax1* and control line B102-1-2 (Fig. 7). Moreover, the analysis of secondary structure further agreed with the results mentioned above. In particular, the β-sheet contents in lines Mut1Ax1-2 and -6 were not significantly different from those in the control line B102-1-2 (Fig. 8). These results helped to explain that the two transgenic lines expressing *mut1Ax1* (Mut1Ax1-2 and -6) had lower transgene expression but had the roughly similar dough mixing properties as those of the control line B102-1-2 (Fig. 3). With respect to all the mutant lines, they were different from the mixing behaviours of line over-expressing *1Dx5* ^42^, in which the peak time increased along with decreases in all other mixing characteristics. This result may be due to the difference in the expression levels of transgene *mut1Ax1* and transgene *1Dx5*; another possible reason is that subunit 1Dx5 originally appeared together with subunit 1Dy10.

From the abovementioned results and the fundamental mechanism regarding the formation of gluten viscoelasticity^34^, it was deduced here that an extra cysteine residue in subunit Mut1Ax1 took part in the cross-linking among glutenin proteins through intermolecular disulfide bonds, which increased the SS contents of gluten and glutenin macropolymers and the size of GMP particles, increased the gluten index and modified the glutenin matrix network of dough, and ultimately improved the dough strength (Figs 3–8). In addition, our study provides an applicable method for exploring the possible mechanisms behind the superiority of subunit pair 1Dx5 + 1Dy10 in comparison with pair Mut1Dx5 + 1Dy10, with subunit Mut1Dx5 without the extra cysteine residue.

## Materials and Methods

### Site-directed mutagenesis and vector construction

Gene *mut1Ax1* was generated from a combination with one part obtained through chemical synthesis (SunBio Medical Biotechnology Ltd., Shanghai, China) and the other part cut from the plasmid pHMW1Ax1 reported by Halford *et al*.^43^.

As shown in Supplementary Fig. S1A, a 564 bp fragment (*a*) was artificially synthesised with the cytosine at the 344 bp position of gene *1Ax1* substituted by guanine, while the restriction sites *Bam*HI and *Nco*I were placed on the sides of its 5′ and 3′ ends, respectively. The corresponding encoding domain contained site-directed mutagenesis that substituted one specific serine codon by a cysteine codon in the repetitive domain. The other linear fragment (*b*) of 1954 bp was gained by digesting the plasmid pHMW1Ax1 with *Nco*I and *Pst*I. Then, fragments *a* and *b* were digested with *Nco*I; subsequent ligation made the whole gene *mut1Ax1* with the restriction sites *Bam*HI and *Pst*I at 5′ and 3′ ends, respectively. Finally, the *mut1Ax1* gene was cloned into pLRPT, which was used as the eukaryotic expression vector^22, 44^. The construct was named pLRPT-Glu-Mut1Ax1. The recombinant vector carrying *wt1Ax1* was constructed in a similar way and named pLRPT-Glu-WT1Ax1 (Supplementary Fig. S1B).

### Wheat transformation and plant regeneration

Common wheat variety L88-31 is a spring wheat with the HMW-GS pair 1Bx17 + 1By18^45^. Due to it being null at the *Glu-A1* locus, this variety was used for particle bombardment in this study.

Genetic transformation started in May 2013. Immature scutella were isolated from young seeds of wheat cv. L88-31 as reported previously^46^. These young seeds were rinsed with 70% (v/v) ethanol for 5 min in a sterile conical flask, subsequently sterilised with 0.1% (v/v) HgCl_2_ for 5 min and rinsed with sterilised deionised water 5 times for 1 min each time. After sterilisation, these isolated scutella were bombarded with the plasmid pLRPT-Glu-Mut1Ax1 or pLRPT-Glu-WT1Ax1 as described previously^47^. T_0_ plants regenerated via *in vitro* tissue culture and then grown to maturity under the same greenhouse conditions as described by Ma *et al*.^28^.

### Identification of transgene integration and expression in wheat plants

To identify the transgenes in transgenic wheat plants, the primers used for PCR amplification were designed according to the sequence of the *CaMV35S* terminator, as its presence in the recombined construct was unique compared with the common wheat genome^23^. The offspring of these transgenic T_0_ plants were subsequently analysed by Southern blotting to confirm whether they were independent transgenic lines. Genomic DNA was extracted as previously reported^48^. After genomic DNA was digested with *Bam*HI, which cuts once within the reconstruction, it was electrophoresed to fully sequence DNA fragments and transfer DNA as previously reported^49^. Afterwards, probe labelling, hybridisation and chemiluminescent detection were performed following the procedure of the DIG High Prime DNA Labelling and Detection Starter Kit II (Roche Diagnostics Gmbh, Mannheim, Germany). Southern blotting pre-hybridisation in this study was carried out at 42 °C for 0.5 h and then hybridisation at 48 °C for 12-14 h. Primers that were used for PCR amplification to prepare the probe were designed with pLRPT-Glu-Mut1Ax1 as the DNA template and produced a 421 bp fragment for DIG labeling^50^.

With respect to T_1_ transgenic plants, developmental seeds of each line were collected at 15 days post anthesis (dpa) and were used to extract the total RNA using a Plant Total RNA kit (Zoman Bio., Beijing, China) (Supplementary Fig. S2A). After treatment with gDNase, purified mRNA was used to synthesise cDNA using the FastQuant RT Kit (Tiangen, Beijing, China). Then, a DNA fragment containing the mutant site was amplified from the synthesised cDNA. A specific primer pair (forward primer: 5′-ATgACTAAgCggTTggTTCTT-3′; reverse primer: 5′-CggAgAAgTTgggTAgTACCCTgC-3′) was designed for transgene *mut1Ax1* or *wt1Ax1* and used for RT-PCR analysis (Supplementary Fig. S2B). The transgene expression in wheat plants was also identified in single half-grains by SDS-PAGE.

### Analysis of transgenic wheat lines and field trials

In the following generations, SDS-PAGE was used to screen transgenic plants expressing *mut1Ax1* or *wt1Ax1* in order to obtain the homozygous transgenic lines that expressed the transgene stably. To compare with the *mut1Ax1* transgenic wheat lines, the *wt1Ax1* transgenic wheat line obtained in this study and line B102-1-2 were used as positive controls. B102-1-2 was provided by Rothamsted Research Institute, UK, and was transformed with plasmids pHMW1Ax1 and pAHC25 in the genetic background of wheat line L88-31^50^. Wheat line L88-31 and non-transgenic segregant line were selected as negative controls.

A field experiment of homozygous wheat lines with a randomised complete block design with two replicates, according to Barro *et al*.^25^, was carried out in October 2015 in Wuhan, China. Plant height, spike length, number of spikelets per spike, number of seeds per spike and 1,000-seed weight were determined. Finally, seeds from all pure lines were harvested in June 2016 and then subjected to subsequent analyses.

### Seed storage protein characterisation and SDS-PAGE analysis

Total seed protein was extracted from flour samples of transgenic and control wheat lines and separated by SDS-PAGE using 10% polyacrylamide gel as described by He *et al*.^51^. Glutenins from 10 mg flour samples were identified by SDS-PAGE in triplicate and were analysed using the densitometry method^2^. To quantify the different storage protein fractions in each line, glutenins and gliadins were extracted from whole grains as reported by Leόn *et al*.^16^. Then, the contents of glutenins and gliadins were determined according to Bradford^52^.

### Measurement of flour protein content and dough mixing and preparation of gluten samples

Grains of two plots per line were blended together, sun-dried and tempered at a 14% (w/w) moisture level for 18 h at room temperature. Subsequently, wheat grains of each line were milled in a Chopin CD1 mill (Chopin technology co., Villeneuve-la-Garenne Cedex, France). The moisture content was determined according to International Association for Cereal Chemistry standard no.110/1 (ICC, 1976). The flour protein content was calculated from the nitrogen content by the method of Dumas according to ICC standard no.167 (%N × 5.7; ICC, 2000), using a Vario Micro cube with two replicates (Elementar co., Hanau, Germany).

A 10-g mixograph (National Manufacturing Co., Lincoln NE, USA) was used for mixograph analysis as described previously^53^. Mixograms and eight key mixing parameters highly related to the flour functional properties were measured and collected in triplicate. The parameters included were MT (min), TmaxBW (min), RBD (%), BWBD (%), PR (arbitrary units, AU), BWPR (AU), MBW (AU) and MTxI (AU). Dough samples mixed to peak were collected in this study and then freeze-dried for SEM analysis.

Gluten samples were obtained according to Popineau *et al*.^42^ with some modifications. Once mixing was finished as mentioned above, dough was quickly scraped off from the mixing bowl and suspended in 140 mL of distilled water by gentle magnetic stirring. After centrifugation at 5,000 g for 15 min, gluten was collected from the top layer of the mixture and then washed until no starch was left. Subsequently, the gluten was freeze-dried, ground into dry powder in a mortar, sealed and stored at 4 °C to determine the contents of free sulfhydryl and disulfide.

### Determination of the gluten index and SDS-sedimentation volume

The gluten index was determined using a Glutomatic 2200 (Perten Instruments Ltd., Hägersten, Sweden) following the America Association of Cereal Chemists, approved method 38-12 A (AACC, 2000). Each sample was performed in quadruplicate. SDS-sedimentation volume test was performed according to AACC international method 56-70.01 (AACC, 1999). Four biological experiments were carried out for each sample.

### Isolation of GMP gel

The gel layer was isolated from wheat flour using the method reported by Mueller *et al*.^41^. To defat, 10 g of flour was mixed with 25 mL of *n*-pentane, magnetically stirred for 30 min at room temperature, and centrifuged at 4,235 g for 20 min at 20 °C (HC-2062, Zonkia, Hefei, China). The subsequent details of the experimental procedure followed that in the report mentioned above. The purified gel was thoroughly lyophilised, briefly ground into dry powder in a mortar, sealed and stored at 4 °C for SE-HPLC analysis.

### GMP particle size distribution analysis

The particle size distribution of GMP was measured using a Mastersizer 3000 (Malvern Instruments Ltd., Worcestershire, UK) with a range of 0.01 ~ 3500 µm. The gel layer was isolated from 2 g of defatted flour as mentioned above. The preparation of GMP dispersions and subsequent details of measurement were carried out according to van Herpen *et al*.^54^. The particle distribution was expressed as the volume percentage. The derived data D[3,2] and D[4,3] were calculated as previously described^55^, which represented the weighted average surface area and the weighted average volume, respectively.

### SE-HPLC analysis

Total proteins were extracted from both transgenic wheat lines and GMP gel samples of each line^56^. A 20 μL supernatant was filtered through a 0.45 μm Nylon membrane, manually injected into the Waters 1525 binary HPLC pump, and fractionated on a Phenomenex Biosep-SEC-s4000 column for 20 min (0.5 mL min^−1^ flow rate). It was further detected at 214 nm with a Waters 2998 photodiode array detector (Waters Corp., MA, USA). As shown in Fig. 4, each eluted profile was divided into four fractions, which corresponded to large-sized polymers (F1), medium-sized polymers (F2), small oligomers (F3) and monomeric gliadins and non-gluten proteins (F4)^53^.

### Determination of free sulfhydryl and disulfide contents

SH_free_ and thiol equivalent groups (SH_eq_) were extracted from the flour of each line and from ground gluten samples using Ellman’s reagents [propan-2-ol, 250 mM Tris-HCl buffer (pH 8.5) and 4 g/L 5,5′-dithiobis-2-nitrobenzoic acid in ethanol (5/5/1, v/v/v)]^31^. The contents of both groups were assayed with three replicates. Tinfoil was used throughout the experiment to avoid light as much as possible. The stepwise diluted L-cysteine (1 mM) reacted with Ellman’s reagents under the same conditions as for the samples to generate a standard curve. Then, the concentration of the sample was read from its absorbance corresponding to the standard curve. All other details were consistent with the previous description^57^. The SS content was calculated as follows: SH_eq_ = 2SS + SH_free_. All values were expressed in μmol g^−1^ protein.

### Scanning electron microscopy

The microstructure of dough mixed to peak was determined by a previously described method^40^. The freeze-dried dough samples of all lines were orderly deposited on a silicon wafer. After gold spraying, the samples were imaged on a Hitachi SEM-600 (Hitachi High-Technologies Corp., Tokyo, Japan) at 1.5 K (15 kV) magnifications.

### Fourier transform infrared spectroscopy

FT-IR spectroscopy has been used to estimate the conformation and conformational changes of proteins^58, 59^. To test the changes in protein secondary structure of dough mixed to peak, a Bruker VERTEX-70 FTIR spectrometer (Bruker Optics, Ettlingen, Germany) equipped with a universal attenuated total reflectance (ATR) accessory was used to record spectra in the 4,000–500 cm^−1^ region at a resolution of 4 cm^−1^ and with 64 accumulated scans^32^. This experiment was performed as previously described^56^.

### Statistical analysis

Primers design and DNA sequences alignment were performed by Vector NTI Advance 11.5.1. SPSS for Windows 16.0 statistical software (SPSS Inc., Chicago, IL, USA) was used for statistical evaluation. Analysis of variance was performed with one-way analysis of variance (ANOVA), and Fisher’s Least Significant Difference (LSD) was used to determine significant differences in agronomic performance among lines and the contents of glutenins and gliadins. The statistical significance for other parameters was determined using Student’s t test.

## Electronic supplementary material

Supplementary information
