# Supplementary material for: Effect of extra cysteine residue of new mutant 1Ax1 subunit on the functional properties of common wheat
Source: Sci Rep. 2017 Aug 8;7:7510. doi: 10.1038/s41598-017-07541-w (PMC5548925; doi:10.1038/s41598-017-07541-w)
Supplement: Supplementary file 1 — Supplementary information [file 41598_2017_7541_MOESM1_ESM.pdf]

# Effect of extra cysteine residue of new mutant 1Ax1 subunit on the functional properties of common wheat

**Miao Li[1,*], Yaqiong Wang[1,*], Fengyun Ma[2,*], Jian Zeng[1], Junli Chang[1], Mingjie Chen[1], Kexiu Li[1], Guangxiao Yang[1], Yuesheng Wang[1,**], Guangyuan He[1,**]**

[1]The Genetic Engineering International Cooperation Base of Chinese Ministry of Science and Technology, The Key Laboratory of Molecular Biophysics of Chinese Ministry of Education, College of Life Science and Technology, Huazhong University of Science & Technology, Wuhan 430074, China

[2]Current Address: Department of Horticulture and Crop Science, Ohio State University, 1680 Madison Avenue, Wooster, OH 44691, USA

* These authors contributed equally.

** Corresponding authors: Yuesheng Wang, Guangyuan He
E-mails: wysh@hust.edu.cn, hegy@hust.edu.cn

Tel: 0086-27-87792271
Fax: 0086-27-87792272

Miao Li* (limiaohust@163.com)
Yaqiong Wang* (wangyaqiongyou@163.com)
Fengyun Ma* (ma.1036@osu.edu)
Jian Zeng (zengjian@hust.edu.cn)
Junli Chang (cjl@hust.edu.cn)
Mingjie Chen (cmj@hust.edu.cn)
Kexiu Li (651380086@qq.com)
Guangxiao Yang (ygx@hust.edu.cn)
Yuesheng Wang** (wysh@hust.edu.cn)
Guangyuan He** (hegy@hust.edu.cn)

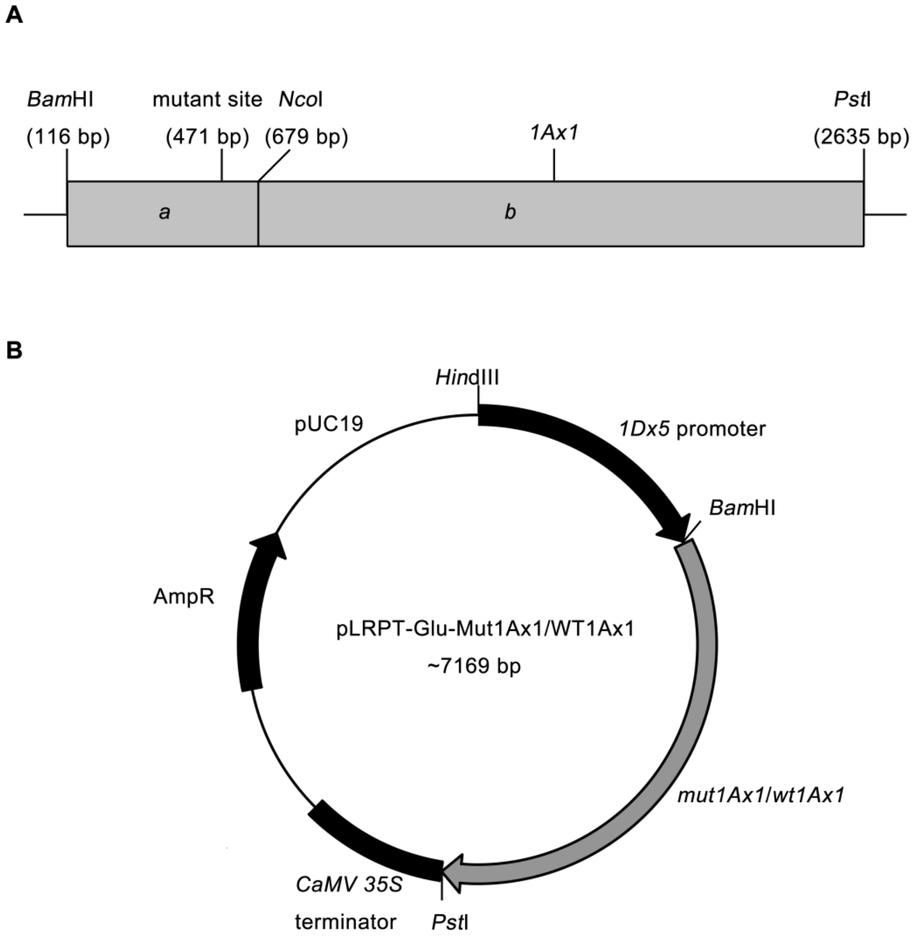


**Figure S1. Schematic diagrams of the *mut1Ax1* gene and plasmid pLRPT-Glu-Mut1Ax1/WT1Ax1 with the position of relevant restriction sites.** (**A**) The *mut1Ax1* gene was generated from a combination with the one part (*a*) obtained from chemical synthesis and another part (*b*) cut from the plasmid pHMW1Ax1. (**B**) The *mut1Ax1* or *wt1Ax1* gene was inserted between the endosperm-specific *1Dx5* promoter and the *CaMV35S* terminator.

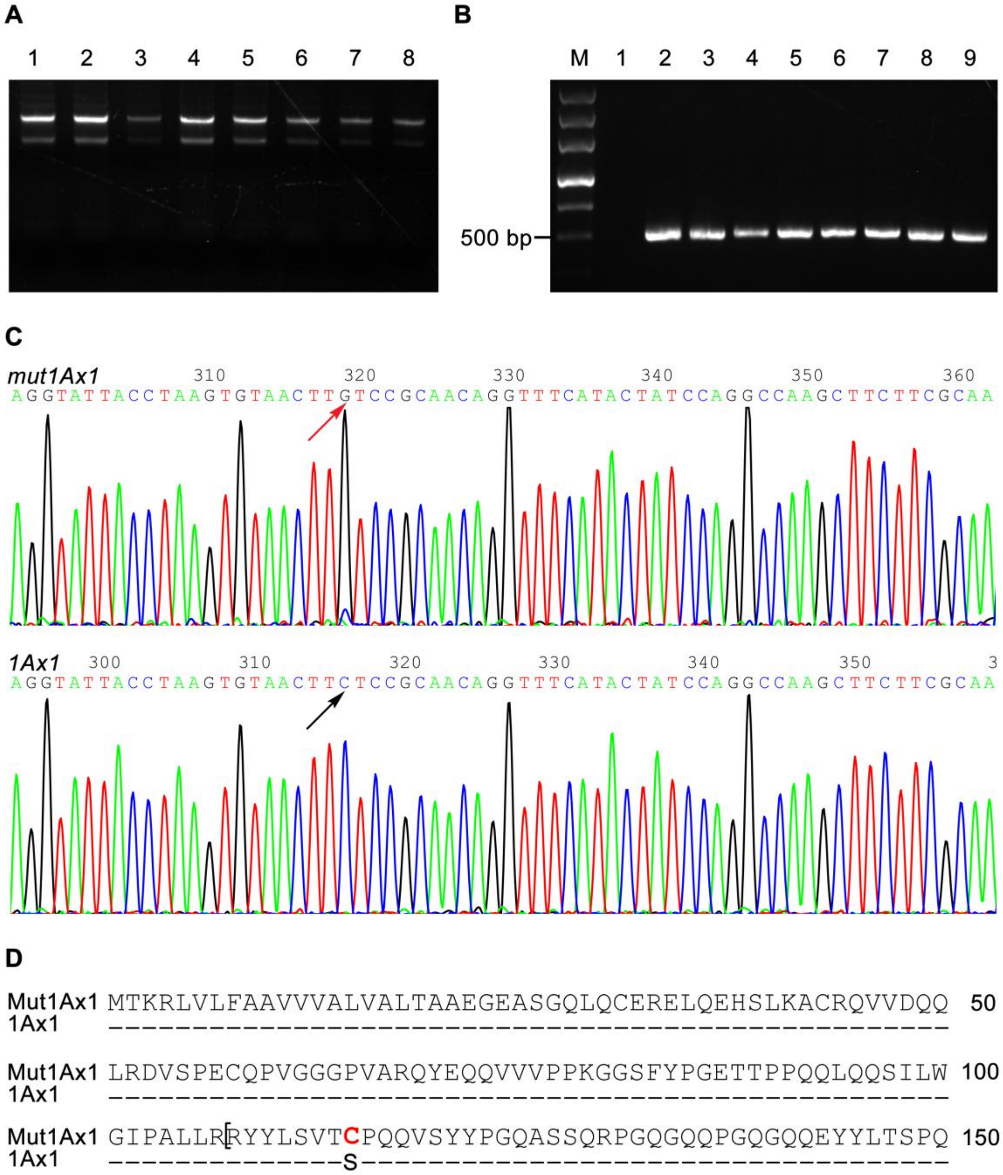


**Figure S2. RT-PCR analysis of transgenic lines expressing *mut1Ax1* and *wt1Ax1* and partial comparison of the derived amino acid sequence of Mut1Ax1 with 1Ax1.** (**A**) Identification for the integrity of mRNA extracted from seeds (15 dpa) of $T_1$ transgenic lines. (**B**) RT-PCR and (**C**) sequencing analyses of transgenic lines. (**D**) Partial comparison of the derived amino acid sequence of Mut1Ax1 with 1Ax1. The mutant site is indicated by the red arrow (**C**) and the red box (**D**).

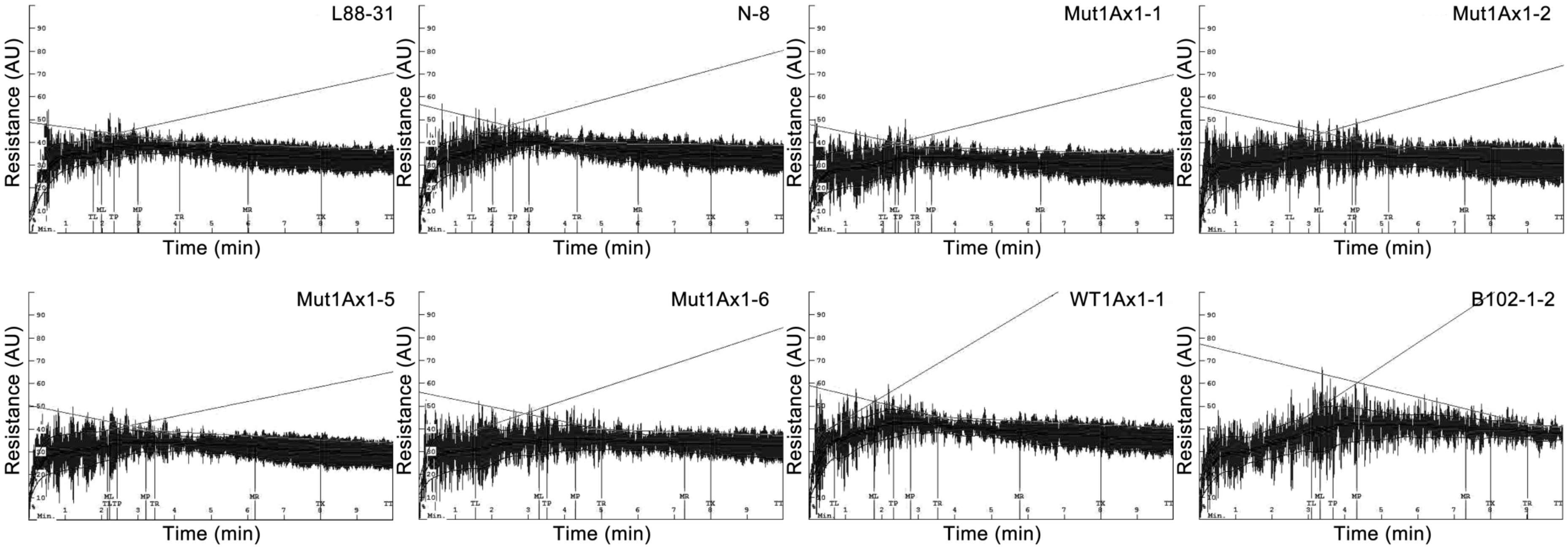


**Figure S3. Dough sample mixing curves of transgenic and control wheat lines.**

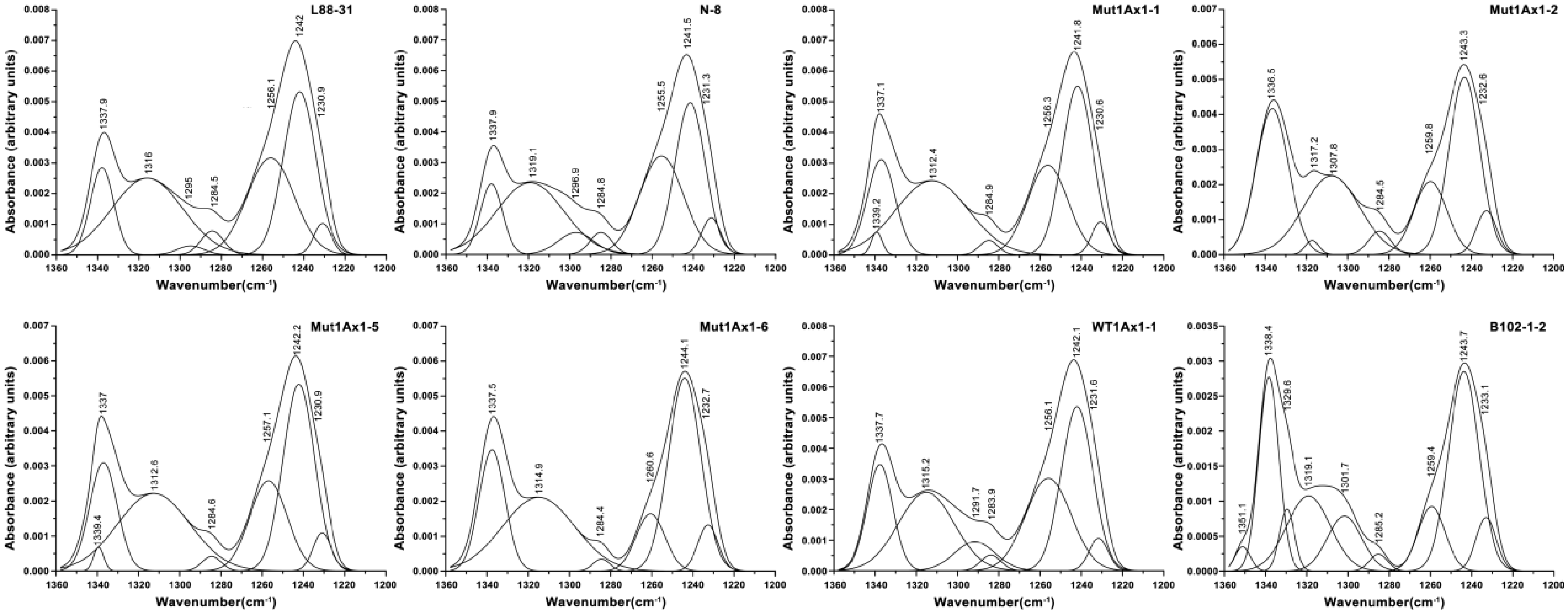


**Figure S4. FT-IR spectra in the 1,360 – 1,200 cm$^{-1}$ range for dough samples of the transgenic and control wheat lines.**

| Parameters | Lines | | | | | | | |
|---|---|---|---|---|---|---|---|---|
| | **L88-31** | **N-8** | **Mut1Ax1-1** | **Mut1Ax1-2** | **Mut1Ax1-5** | **Mut1Ax1-6** | **WT1Ax1-1** | **B102-1-2** |
| Plant height (cm) | 101.8abABC | 96.4cC | 103.2abABC | 102.8abABC | 107.1aA | 100.5bcABC | 104.6abAB | 99.5bcBC |
| Heading date (days) | 161.2 | 163.4 | 163.4 | 161.7 | 162.4 | 163.5 | 163.8 | 161.4 |
| Growth period (days) | 210.5abA | 211.8aA | 206.8bcAB | 210.3abA | 207bcAB | 209.8abA | 212aA | 204cB |
| No. of spikelets per spike | 18.8 | 20.4 | 19.9 | 19.4 | 20.4 | 19.8 | 20.1 | 19.8 |
| No. of seeds per spike | 42.85bB | 44.1bB | 52.1aAB | 43.8bB | 53.3aAB | 56.6aA | 49.4abAB | 43.6bB |
| 1,000-seed weight (g) | 26.8 | 27.7 | 27.0 | 26.8 | 27.8 | 27.6 | 27.2 | 27.7 |

**Table S1 Agronomic performance of transgenic and control wheat lines.** Values within the same parameter followed by the same letter are not significantly different at the 0.05 (small letter) and 0.01 (capital letter) probability levels. Each average was calculated from fifteen biological experiments.

| Parameters | Lines | | | | | | | |
|---|---|---|---|---|---|---|---|---|
| | **L88-31** | **N-8** | **Mut1Ax1-1** | **Mut1Ax1-2** | **Mut1Ax1-5** | **Mut1Ax1-6** | **WT1Ax1-1** | **B102-1-2** |
| Endogenous HMW | **17+18** | **17+18** | **17+18** | **17+18** | **17+18** | **17+18** | **17+18** | **17+18** |
| Transgenic HMW | NA | NA | Mut1Ax1 | Mut1Ax1 | Mut1Ax1 | Mut1Ax1 | 1Ax1 | 1Ax1 |
| *Protein characterisation* | | | | | | | | |
| Flour protein content (%)[b] | 7.52 | 7.89 | 8.27 | 8.21 | 8.21 | 8.72 | 8.46 | 9.29 |
| Glutenin (μg $mg^{-1}$ flour)[c] | 3.55cC | 3.53cC | 3.82bcBC | 4.01abAB | 3.69cC | 4.07aAB | 3.62cC | 4.19aA |
| Gliadin (μg $mg^{-1}$ flour)[c] | 4.97 | 4.91 | 4.41 | 4.67 | 4.63 | 4.76 | 4.75 | 4.67 |
| Glutenin/gliadin | 0.74bBC | 0.72bC | 0.87aAB | 0.86aAB | 0.8abABC | 0.86aAB | 0.75bBC | 0.9aA |
| Gluten index[d] | 44.21eE | 44.56eE | 53.4cdCD | 59.47bcBC | 53.7cdCD | 63.51abAB | 50.21deDE | 67.99aA |
| SDS sedimentation (ml)[e] | 12.53eD | 12.87eD | 16.13cC | 15.27cdC | 16.1cC | 14.97dC | 17.6bB | 32.37aA |

**Table S2 Comparisons of flour quality-related parameters of the transgenic and control wheat lines[a].** NA, not applicable. [a] Results are expressed based on 14% moisture. [b] Protein contents were determined by the Dumas method with an average of 2 replications. [c] Glutenins and gliadins were determined using the Bradford assay with an average of 4 replications. [d] Gluten index was determined using AACC method 38-12A with an average of 4 replications. [e] SDS-sedimentation volume was determined according to AACC international method 56-70.01 with an average of 4 replications. Values within the same parameter followed by the same letter are not significantly different at the 0.05 (small letter) and 0.01 (capital letter) probability levels.
